# The Mindfulness Map: A Practical Classification Framework of Mindfulness Practices, Associated Intentions, and Experiential Understandings

**DOI:** 10.3389/fpsyg.2021.727857

**Published:** 2021-10-12

**Authors:** Nava Levit-Binnun, Keren Arbel, Dusana Dorjee

**Affiliations:** ^1^Muda Institute for Mindfulness, Science and Society, Sagol Center for Brain and Mind, The Interdisciplinary Center (IDC), Herzliya, Israel; ^2^Department of East Asian Studies, Tel Aviv University, Tel Aviv-Yafo, Israel; ^3^Psychology in Education Research Centre, Department of Education, University of York, York, United Kingdom

**Keywords:** mindfulness, compassion, loving kindness, map, framework, experiential understanding, intention, insight

## Abstract

When considering the numerous mindfulness-based and mindfulness-informed programs that have flourished in the past decades it is not always clear that they all refer to the same “mindfulness. ” To facilitate more clarity and precision in describing, researching and teaching mindfulness in the secular settings, we propose a classification framework of mindfulness practices, intentions behind them and the experiential understandings the practices may aim to develop. Accordingly, the proposed framework, called the Mindfulness Map, has two axes. The first axis outlines mindfulness practices (and associated instructions) classified into four groups (MGs), e.g. the MG1 focuses on cultivating attention to the present moment somatic and sensory experience while the MG4 focuses on cultivating the ability to recognize and deconstruct perceptual, cognitive and emotional experiences and biases. The second axis outlines possible intentions (INTs) to cultivate particular experiential understanding (EU) *via* teaching and practicing the MGs, e.g., the INT1 designates the intention to gain EU of how our relationship to experience contributes to wellbeing, the INT2 refers to the intention to gain EU of the changing nature of body, mind and external phenomenon. We suggest that the same MG can lead to different EUs outcomes based on the specific INTs applied in their teaching or practice. The range of INTs and EUs included here is not exhaustive, there are further types the Map could be expanded toward. Aside from encouraging more fine-grained distinctions of mindfulness practices, the proposed Map aims to open discussions about interactions between MGs, INTs, EUs and practice outcomes. The Map may facilitate more nuanced and precise approaches to researching the range of outcomes cultivated by mindfulness practices, help bridge contradictory findings, and catalyze further debate and research into ethical aspects of mindfulness. The Map also highlights the need for further teaching development and research on longer-term trajectories of mindfulness practice. While the proposed Mindfulness Map organises the mindfulness practice territory along two axes, it is aimed as a starting point for further discussion and can be further revised and/or expanded by other axes.

## Introduction

“Mindfulness” is currently often used as an umbrella term. It typically denotes practices involving paying attention to both external and internal bodily sensations or mental contents, with certain attitudes and intentions. Mindfulness is also sometimes considered as a process, a state of mind and/or a trait (Davidson and Kaszniak, 2015; Dorjee, 2017). While the working definition that was coined by John Kabat-Zinn (“the awareness that emerges through paying attention on purpose, in the present moment, and non-judgmentally to the unfolding of experience moment by moment,” (Kabat-Zinn, 2003) is often found in the non-Buddhist contexts (Kabat-Zinn, 2005), it is generally accepted that the definition is not exhaustive or exclusive (e.g., Bishop et al., 2004; Brown and Ryan, 2004; Fletcher and Hayes, 2005; Dorjee, 2010; Chiesa, 2013; Nilsson and Kazemi, 2016). Similarly, in the Buddhist tradition there is not a single agreed definition, theory or understanding of the term “mindfulness” (e.g., Dorjee, 2010; Bodhi, 2011; Dunne, 2011, 2015; Gethin, 2011, 2015; Anālayo, 2016).

Historically, mindfulness practices were considered foundational for the contemplative life and typically practiced alongside other practices that strengthen wholesome qualities, such as kindness and compassion, or in preparation and support for meditations that generate insight into the nature of the self (Dorjee, 2017; Dahl and Davidson, 2019). Arguably, psychological processes such as non-reactive attention and associated metacognitive (introspective) skills cultivated through mindfulness are involved in and built upon in other forms of meditation including those cultivating virtue-orientation and self-inquiry (Dorjee, 2013; Dahl and Davidson, 2019). This wide-reaching applicability of mindfulness across meditation types may be one of the reasons why the term “mindfulness” is now often used in a way that includes or implies other meditation practices such as those developing compassion or insight.

Thus not surprisingly, when looking at the myriad of mindfulness practices and programmes with mindfulness elements that have flourished in the past 30 years (Harrington and Dunne, 2015), it is often not clear whether they all refer to the same “mindfulness.” For example, even within the well-researched Mindfulness-Based Stress Reduction (MBSR, Kabat-Zinn, 2013) and Mindfulness-Based Cognitive Therapy (MBCT, Segal et al., 2013) programs, participants learn mindfulness in various ways, across a range of practices such as mindful eating, body-scan, mindful yoga, and sitting meditations ranging from awareness to breathing to practicing open awareness (Kabat-Zinn, 2013; Segal et al., 2013). Recently, Thupten (2019), commented that: “Complications arise when one starts examining what exactly is being taught as ‘mindfulness' in the secular context. Some see focus and awareness to be the two main skills that are taught; others emphasize open awareness and the attitude of non-judgment; and some also bring the affective tone of tenderness or kindness to be part of the core practice. Many modern mindfulness teachers also include elements of loving-kindness as part of their instruction.”

Over the past years, several authors have proposed models and classifications of contemplative practices (Dorjee, 2010; Vago and David, 2012; Schmidt, 2014; Dahl et al., 2015; Garland et al., 2015; Lutz et al., 2015; Grossenbacher and Quaglia, 2017; Khoury et al., 2017; Lindsay and Creswell, 2017; Fresco and Mennin, 2019) usually emphasizing the underlying cognitive mechanisms. The present paper continues these efforts with the aim to reduce confusion and increase precision in describing and differentiating practices that are currently associated with the term mindfulness and suggesting a two-dimensional classification map. The first axis of the Mindfulness Map outlines four **groups of mindfulness practices (MGs)** commonly found in contemporary (secular) scientific discourse on mindfulness.

A related challenge is the lack of clarity about the experiential understandings that can be cultivated in different practices and the associated intentions behind the practices. The term “insight” is sometimes used in this context, but we prefer the term “experiential understanding” due to the varied meanings of “insight” in psychology and the Buddhist discourse. We understand experiential understanding interchangeably with the term “modes of existential awareness” (Dorjee, 2016) which describes an overarching phenomenological state associated with our sense of self^1^ and perception of reality (an overarching “optic” of perceiving self and reality), but choose to use the term “experiential understanding” in the context of the current Map because this concept is more intuitively graspable for practitioners and teachers. For instance, some of the mindfulness practices may cultivate experiential understanding of how our relationship to thoughts, emotions and sensations contributes to or undermines our wellbeing. Specifically, we know that ruminative immersion in negative thoughts is symptomatic of anxiety and depression whereas observation of negative thoughts as passing and fleeting events (decentering), which can be considered an example of an experiential understanding, is predictive of recovery from anxiety and depression (e.g., Hoge et al., 2015). We suggest here that an experiential understanding (e.g., of how our relationship to experience relates to wellbeing) can be cultivated to some depth in all mindfulness practices, although some practices are less associated with the intention to do so than others and this may modulate the depth of the resulting experiential understanding. Therefore, the second axis of the Map aims to capture *types of intentions (INTs) towards cultivation of particular experiential understandings* (EUs) that can be developed through mindfulness practices. In this way, the same mindfulness practice can result in different EUs based on the INTs the teacher and/or the practitioner brings to the practice. The postulation of the INT dimension can open focused discussions about the unintentional and intentional types of EUs cultivated through mindfulness practices. It can also support further considerations about the relevance of intentions for cultivating ethical behaviors to different mindfulness-based programs and interventions. While we identified four MGs, there are likely more types of INTs for EUs than those proposed in the Mindfulness Map. Notably, the proposed Map distinguishes INTs for EUs from reasons for mindfulness practice. The reasons can range from stress reduction, through health-focused clinical reasons, such as reducing anxiety symptoms or chronic pain, skill-enhancement for improved academic or workplace performance to self-exploration and better self-understanding (Shapiro, 1992; Pepping et al., 2016; Sparby and Ott, 2018).

Importantly, the proposed mindfulness classification map is trying to capture the variety of current uses of the term “mindfulness” in secular contemplative teaching and scientific discourse; it does not aim to describe the full traditional Buddhist landscape of practices, EUs and associated INTs. The primary aim of the classification proposal presented here is to provide a practical framework that can support mindfulness teachers, practitioners and researchers to clearly locate the particular mindfulness they teach, practice or research within a territory of MGs, associated INTs and EUs. In this way, the Map may facilitate development of a much-needed longer-term perspective on both mindfulness practice and teaching, beyond MBSR or MBCT courses (Dorjee, 2017). It may also support a finer classification and specification of mindfulness practices in mindfulness research, possibly leading to more comprehensive understanding of mindfulness, its mechanisms, and effects. Markedly, the proposed Map is aimed as a starting point for further discussion and may be revised and/or expanded by other axes. In what follows we describe the four MGs and then give examples of four types of INTs. We then elaborate on how the different types of EUs manifest in each combination of MGs and INTs.

## The Four Groups of Mindfulness Practice

The four mindfulness practice groups (MG) presented in the Mindfulness Map were inspired by Kristin Neff's work (2015)^2^ on four aspects of mindfulness practice: (m1) paying attention to experience in the present moment; (m2) relating to experience without judgment or resistance; (m3) Relating to the experiencer with the desire to alleviate suffering (compassion); (m4) understanding better the nature of both experience and the experiencer (wisdom). We adopted the four-fold division with some alterations and called the categories *mindfulness practice groups (MG)*, because each of them is not a description of a single technique, but a grouping of several techniques with similar key instruction characteristics. In addition, we altered the definitions in several cases to be consistent with terms used in the psychological literature.

The Mindfulness practice Groups (MGs) are:

MG1—Cultivating^3^ attention to the present moment somatic and sensory experience
MG2—Cultivating non-reactive and a non-judgmental attitude to experience
MG3 –Cultivating wholesome and pro-social mental habits
MG4—Cultivating the ability to recognize and deconstruct perceptual, cognitive and affective experiences and biases.

The descriptions below cover the main characteristics of techniques and instructions in each mindfulness group.

### MG_1_: Cultivating Attention to the Present Moment Somatic and Sensory Experience

Practices in MG1 strengthen concentration capabilities and stabilize awareness of somatic and sensory experience. The ability to observe the present bodily experience (as opposed to analyzing or conceptualizing it) is developed through MG1 techniques.

For example, MG1 includes practices that instruct practitioners to attend to their breath and/or body without additional instructions (e.g., Arch and Craske, 2006; Zeidan et al., 2010a). Other practices in this family would be listening to sounds, observing visual, tactile, olfactory, or gustatory objects (e.g., raisin tasting exercise), variations of body-scan, mindful running, and certain yoga practices (Petrillo et al., 2009; Kabat-Zinn, 2013; Hong et al., 2014; Schultchen et al., 2019). An informal mindfulness practice of MG1 would be attending to everyday activities like washing the dishes or eating a meal—with particular emphasis on the importance of noticing sensory experience as it occurs in the present moment (Hanley et al., 2015).

Mindfulness practices that have been employed in studies that investigated the effects of brief mindfulness practices often belong to MG1 (Arch and Craske, 2006; Dickenson et al., 2013). For example, in Schindler et al., 2019 (p. 1057), “Participants in the mindfulness exercise condition listened to a 5-min recording about concentrating on one's breath and becoming aware of what is happening in the present moment.” Typical brief MG1 instructions ask participants to: “focus on the actual sensations of breath entering and leaving the body. There is no need to think about the breath—just experience the sensations of it. When you notice that your awareness is no longer on the breath, gently bring your awareness back to the sensations of breathing” (Arch and Craske, 2006, p. 1852).

MG1-type practices have been shown to benefit wellbeing and mental health, likely through stabilization of awareness and reduction in rumination. This has been assessed with indexes of mood, cardiovascular function, affect, aggression, attention, pain, social stress responses, mental state attribution and empathy (e.g., Zeidan et al., 2010a,b; Dickenson et al., 2013; Creswell and Lindsay, 2014; Tan et al., 2014; Norris et al., 2018).

### MG_2_: Cultivating a Non-reactive and Non-judgmental Attitude to Experience

In MG2 practices, the attitude of non-judgmental acceptance is added to the MG1 practices and one cultivates an interested and non-reactive stance toward present moment experience which now also includes mental phenomena. On top of the ability to focus on the elements that make up sensory and somatic experiences (MG1), one is asked to actively allow the experience to unfold without self-criticism or denial of unwanted sensations, emotions or cognition. This includes non-reactive non-judgmental noticing of automatic and habitual tendencies of evaluating, craving, rejecting, denying and avoiding. These attitudinal qualities that are now added to MG1 practices create a sense of space between the object of observation and the automatic reaction. This in turn, facilitates the weakening of automatic reactions as one familiarizes oneself with a different way of responding to experience including the attitudes of acceptance, curiosity, and openness. Practitioners are guided to distinguish between the elements that make up experience (sensations, thoughts, urges) and the reactions to these elements, then carefully observe the dynamic relations between them moment by moment.

Importantly, while MG1 is more focused on stabilizing awareness on an anchor (e.g., the breath), in MG2 and onward, the *attitude* toward our experience is a key aspect to develop. As a curious and non-judgmental attitude is cultivated in MG2, it can be directed to a various anchor of attention, allowing the practice to generalize inside and outside of formal mindfulness practice. This is a crucial transition as it enables more frequent experiences of mindful states, that can, in time, induce trait-like shifts in relating to our experience and to others.

The very first practices in MBSR and MBCT programs (e.g., body scan, movement practices) begin as MG1 but *via* further instructions to cultivate the attitudinal qualities they shift into MG2. In the beginning these attitudes are geared toward sensory and somatic experiences. Later practices (e.g., sitting meditations) in these programs, employ a similar attitude toward affective and cognitive states (Segal et al., 2013).

It seems that most mindfulness research to date has focused on the MG2 practices. Studies of short interventions often employ basic MG2 practices that add to MG1 a non-judgmental and accepting attitude. A typical example is the study of a brief regular mindfulness practice intervention and its effects on cognitive and affective brain indexes in older adults (Malinowski et al., 2017, p. 81) in which participants “were required to focus their attention on the sensations accompanying their breathing, either attending to the experience at the nostrils, around the diaphragm or the movement of the abdomen when inhaling and exhaling, without manipulating the breath in any form. Whenever attention would slip or wander off, the task would be to become aware of it and, without further elaboration, to redirect the focus of attention back to the sensation of breathing. Participants were instructed to recognize other arising thoughts, feelings or sensations, trying not to judge or evaluate them, and maintain a curious, non-elaborating attitude toward them.” Most practices in MBSR/MBCT interventions could be categorized as MG2s (e.g., Alkoby et al., 2019). Beneficial effects of MBSR/MBCT on mental health have been well documented and there is also evidence of these interventions leading to improvements in wellbeing and aspects of cognition (e.g., Alberts and Thewissen, 2011; Gu et al., 2015; Goldberg et al., 2018b; Querstret et al., 2020). Similarly, non-MBSR/MBCT interventions in MG2, often very brief, have been shown to improve aspects of cognition such as attention and inhibitory control (e.g., Malinowski and Shalamanova, 2017; Malinowski et al., 2017; Schöne et al., 2018; Pozuelos et al., 2019).

### MG_3_: Cultivating Wholesome and Pro-Social Mental Habits

MG3 practices aim to explicitly develop wholesome and prosocial emotions, thoughts and behaviour's including kindness, compassion, and appreciation (Shapiro et al., 2006; Dahl and Davidson, 2019; Thupten, 2019). They include practices such as Loving Kindness meditation, Compassion meditation and Gratitude practices and usually involve active use of imagery to evoke particular emotions (e.g., imagining oneself, a benefactor, a neutral person, a difficult person or beings in general and addressing them with phrases of compassion and/or kindness), and explicit cognitive emphasis on appreciating what is wholesome.

At least some of the prosocial qualities, for example kindness, seem to be considered mindfulness-based in the early Buddhist discourse^4^, even though they don't seem to be discussed in this way in the Mahayana schools (Wallace, 1999). Still, various mindfulness practices that stabilize present-centered awareness are typically practiced in preparation or alongside loving-kindness and compassion practices (Dahl and Davidson, 2019). For example, a compassion practice involves sustaining attention on the content of the meditation and employment of non-judgmental attitude when unpleasant sensations, emotions or thoughts arise (e.g., when awareness of suffering increases) (Neff and Dahm, 2015).

Indeed, MG3 practices often appear in programs and in studies in conjunction with mindfulness practices (Brewer et al., 2011). For example, “Mindful Self-Compassion (K. Neff and Germer, 2018) and “Mindfulness-based compassionate living” (Van den Brink and Koster, 2015) are 8-week programs that develop both the skills of mindfulness and compassion. Studies on the effects of these programs or similar programs have found beneficial effects on wellbeing, e.g. increases in positive emotions and self-compassion (Neff and Germer, 2013; Mantzios and Wilson, 2015; Shahar et al., 2015; Friis et al., 2016; Graser et al., 2016; Eriksson et al., 2018; Ondrejkov et al., 2020).

### MG_4_: Cultivating the Ability to Recognize and Deconstruct Perceptual, Cognitive and Emotional Experiences and Biases

In MG4 practices, one deconstructs subjective experience into its various components (attention, sensation, feelings, cognition, and perception) and observes the interplay between them. The curious and non-judgmental attitude to the present experience fostered in MG2 leads to initial experiences of decentering—perceiving mental contents as transient fleeting phenomena (Fresco et al., 2007). Decentering develops further and is built on in MG4 practices supporting deeper self-inquiry. In the modern context of mindfulness-based approaches an example of an initial practice in this group is the “choiceless awareness” practice in MBSR and MBCT whereby one is instructed to simply rest one's awareness on mental components of experience, without identifying or engaging with any particular component (Kabat-Zinn, 1990, pp. 59–74; Segal et al., 2002, pp. 146–147, 164–165). Such opening up of a degree of space between awareness and mental phenomena and between stimulus and response may result in the recognition “that “mind” is not identical to mental phenomena. In other words, we are not our thoughts, feelings, or experiences (Chambers et al., 2009) as is emphasized in the “thoughts are not facts” practice in MBCT (Segal et al., 2013, p. 322). The key difference between MG2 and MG4 practices is the targeted instruction examining the nature of experience and Self in MG4 in contrast to focus on development of attitudinal qualities in MG2. As the ability to deconstruct experience develops, automatic reactivity further weakens and distorted perceptions, misconceptions and biases that underlie craving, avoiding and rejecting leading to mental distress are deconditioned.

MBSR and MBCT don't go beyond the initial MG4 practices associated with decentering and one of the pressing questions in the secular mindfulness field is how the repertoire of secular MG4 could be expanded further toward more advanced MG4 practices. In the traditional Theravada Buddhist context, some of the *vipassana practices* can be included in MG4 (Chiesa, 2010; Dunne, 2015). These could serve as a basis for expanding the MG4 practice in the secular mindfulness context. For example, an advanced MG4 practice would be examining cognitive reification processes that give rise to implicit beliefs and biases that thoughts, emotions, and perceptions are accurate depictions of reality (Dahl et al., 2015). Such practice may, for instance, invite practitioners to sustain a mindfulness level that can notice the “gaps” in the elements of experience (e.g., thoughts, emotions, mind states, pleasant, and unpleasant experiences, the sense of Self) and investigate how noticing the gaps changes their perception and the sense of them being “real” (e.g., Burbea, 2014, p. 95). These practices can be built on in further deeper practices deconstructing the experience of self and examining self as a context (e.g., as suggested in Acceptance Commitment Therapy (ACT), (Fletcher and Hayes, 2005), and seeing that unitary sense of self is constructed by changeable self-related experiences and narratives and therefore is illusory (Dahl et al., 2015, 2020). With time, these insights are internalized, and they become a trait-like perspective on experience, as opposed to a transient state of awareness (transformation from state to trait).

In contemporary scientific literature, vipassana or open monitoring practices are often referred to as advanced mindfulness practices. Moreover, studies of long-term practitioners often refer to a range of traditional Buddhist practitioners as mindfulness meditators creating confusions (e.g., Lykins and Baer, 2009; Chiesa, 2010; Manna et al., 2010; van den Hurk et al., 2010; Chiesa and Malinowski, 2011; Ferrarelli et al., 2013; Ataria et al., 2015; Laneri et al., 2016; Kral et al., 2018). For example, a recent highly cited review of neural correlates of mindfulness made integrative inferences across studies ranging from MBSR, to Vipassana, Zen Buddhism and Dzogchen (Tang et al., 2015). These discrepancies call for more refined classification of advanced mindfulness practices and their overlaps and differences from meditation practices currently labeled as mindfulness.

Importantly, the numerical values associated with the MGs do not necessarily represent a rigid progression, as one may not always begin with MG1 practices and advance through MG2, MG3, and MG4. They represent, however, degrees of complexity in self-inquiry as MG1 practices are simpler and less focused on self-exploration than MG4 practices. To some extent skills and qualities such as non-reactivity and decentering that are developed in MG1 and MG2, are also needed to fully develop MG3 and MG4—but not the other way around.

## The Four Types of Intentions and Associated Experiential Understandings

The practices in the four MGs can be taught and practiced with a variety of intentions (INTs) for EUs which are overarching phenomenological states resulting from meditation practice. We propose that INTs might be essential facilitators of EUs they are directed toward, they may enable continuous engagement with practices cultivating certain EUs, and catalyse further progression onto more refined EUs. It is an empirical question whether some EUs arise regardless of INTs a practitioner or teacher brings to their practice, simply as a result of particular MG practice types. As a starting point, we propose here an outline of the possible INTs. Although there may be a range of INTs, in this paper we chose four specific INTs to demonstrate the second axis of the Map.

The four Intentions (INTs) are:

**INT**_**1**_ Intention to gain experiential understanding of how the relationship to experience contributes to mental distress and wellbeing
**INT**_**2**_ Intention to gain experiential understanding of the changing nature of body, mind and external phenomenon
**INT**_**3**_ Intention to gain experiential understanding of the relationship between sense of self and mental distress and wellbeing
**INT**_**4**_ Intention to gain experiential understanding of how positive and prosocial mental states contribute to wellbeing.

The first three INTs are based on the three traditional Buddhist characteristics of experience (*tilakkha?a)*, that, according to classical Buddhist tradition, are central to understanding the causes of mental distress and alleviating it^5^. These are: (1) *dukkha*, meaning dissatisfaction and suffering; (2) *anicca*, meaning impermanence; and (3) *anatta*, meaning not-self. In addition, we have added an INT that is based on the Buddhist wholesome intentions and states of mind such as “loving kindness,” “compassion” “rejoicing” and “equanimity” (Brahmaviharas). We are focusing on these four INTs for several reasons. First, many secular mindfulness practices are derivatives of programs (e.g., MBSR, ACT) which, according to their founders, are grounded in Buddhist meditation (Hayes, 2002; Kabat-Zinn, 2011; Husgafvel, 2018). Second, these INTs resonate with elements of experience and EUs that are emphasized and explored in the context of secular mindfulness practices and interventions, although not always clearly articulated. For example, according to the Mindfulness Based Interventions: Teaching Assessment Criteria (MBI:TAC, third-version, 2021), skillful facilitators should be able to assist participants in noticing whether elements of experience change or are constant, explore the sensations of reactions/responses to experiences, investigate how bringing awareness and particular attitudes toward experiences affect the experiences, and illuminate how they see the ways in which their mind becomes “caught” or stuck in their particular way of relating to experience (see the Mindfulness Based Interventions: Teaching Assessment Criteria; MBI:TAC, third-version, 2021). In other interventions, such as ACT, elements of experience are investigated in order to gain insights into the nature of the Self (Fletcher and Hayes, 2005).

Importantly, here we distinguish between INTs for EUs and reasons for practicing mindfulness. The reasons can range from fulfillment of spiritual aspirations (Symington and Symington, 2012), toward specific clinical goals such as alleviation of depression or stress symptoms (e.g., MBCT) or toward self-enhancement, (e.g., Mindful Sport Performance Enhancement (MSPE, Kaufman et al., 2009); Mindfulnes-Based Mind Fitness Training (MMFT, Gans et al., 2014; Jha et al., 2015); mindful eating to reduce craving, (Kristeller et al., 2014; Mason et al., 2018). An early study suggested that reasons for meditation practice can change with longer-term practice, progressing from self-enhancement to self-exploration and then self-understanding (Shapiro, 1992). There have been repeated calls for the reasons behind mindfulness practice to be examined empirically (Harrington and Dunne, 2015; Pepping et al., 2016) since they may modulate practice outcomes (Davidson and Kaszniak, 2015; Dorjee, 2016). Here we propose the INT axis as part of the Mindfulness Map to capture intentions that may closely interact with reasons for mindfulness practice but denote a particular experiential perspective with which we approach a mindfulness practice.

Since the focus in this proposal is on mindfulness in the secular context, the INTs are formulated in relation to reducing mental distress and supporting wellbeing. The term *mental distress*, in this context, refers to both mental illness as understood in Western psychology and more subtle forms of mental discomfort which would not be considered pathological but impact wellbeing and are gaining increasing research interest, such as greed (Ryff, 2018) or intention to harm. EUs facilitated by application of INTs are associated with experiential knowledge that plays a key role in reduction of mental distress which can be achieved through application of this EU to managing our thoughts, feelings, behaviors and sense of self. Similarly, *wellbeing* in the context of the above INTs is not simply understood as pleasure-based happiness or life satisfaction as in the subjective wellbeing conceptualizations (e.g., Diener et al., 1985). It is closer to the notion of eudemonic wellbeing (Ryff, 1989) formulated in terms of meaningful life inspired by Aristotelian virtues. Yet, the concept of wellbeing in the current proposal goes beyond psychological eudemonic wellbeing in its grounding of wellbeing in the applied self-inquiry into mental distress and its sustaining in everyday functioning. This understanding of wellbeing arises from observing and relating to our experience (i.e., applying mindfulness), rather than mere intellectual learning about mental distress and its causes.

### Experiential Understandings Resulting From Intentions Applied to the Mindfulness Practice Groups

Each of the four INTs applied to the four MGs leads to different types of resulting experiential understandings (EUs) creating The Mindfulness Map (see Table 1). As can be seen from the table, in most cases, there is a sense of deepening progression of EUs implicit in application of INTs to the four MGs and from MG1 through to MG4.

**Table 1:** The Mindfulness Map.

	**MG1: cultivating intentional attention to the present moment**	**MG2: Cultivating a non-reactive and non-judgmental attitude to experience**	**MG3: Cultivating wholesome and pro-social mental habits**	**MG4: Cultivating the ability to recognize and deconstruct perceptual, cognitive and emotional experiences and biases**
**INT1: to understand how the relationship to experience contributes to mental distress and wellbeing**	Experiential understanding (EU): Beginning to identify the relationship between attention regulation and mental distress.	EU: Mental distress is understood to be dependent on craving and rejecting certain experiences. A non-reactive and non-judgmental attitude creates a space between experience and reaction and hence reduces mental distress.	EU: Discerning that mental distress or wellbeing are conditioned by the mental attitudes present at each moment. Experiencing internal or external experiences with the presence of wholesome states reduces mental suffering, while the presence of unwholesome states increases it.	EU: Understanding of the relationship between lessening of mental distress and reduced craving, avoiding/rejecting is deepened following cultivation of the decentering from experience (stepping out from thoughts, emotions, sensations).
**INT2: to understand the changing nature of body, mind and external phenomena**	EU: Beginning to observe the dynamic changing nature of the mental processes, particularly those including somatic and sensory experiences.	EU: Beginning to understand that all the elements that make up experience (sensations, thoughts, emotions, urges) are interdependent, transient and temporary occurrences.	EU: Seeing that wholesome states are also transient and dependent on various causes and conditions.	EU: Deepening and internalizing the understanding that every experience, as well as every external phenomena (e.g., property, people, etc.) is transient, temporary and interdependent in its occurrence. This facilitates the release of craving and identification from them.
**INT3: to understand the relationship between sense of self and mental distress and wellbeing**	EU: Beginning to recognize a lack of control over somatic and sensory experiences. This can start to reduce the rigidity of sense of self.	EU: Beginning to understand how craving and avoidance/rejection patterns solidify the sense of self; beginning to understand the relation between degrees of solidification in the sense of self, and the reduction in mental distress.	EU: Identifying the relationship between self-modes (e.g., flexible or rigid sense of self) and the presence of wholesome or unwholesome states (cognitions, attitudes and emotions). Beginning to see that during wholesome states, the “self,” “other” and the “world” are less rigidly separated than previously assumed.	EU: Understanding that the “self” is a complex phenomenon constructed from changing and interdependent processes. It is not static, solid and independent, but rather depends on an ever-changing context.
**INT4: to understand how positive and prosocial mental states contribute to wellbeing**	EU: Not realized in MG1.	EU: Reducing reactivity allows for the acceptance of the present experience resulting in more acceptance of oneself and others and consequently lesser distress.	EU: Actively cultivating wholesome states and pro-social habits (which are more than cultivating acceptance and self-compassion as in MG2) enables the realization how these states support and enhance wellbeing.	EU: The weakening of identification with experience and deepened understanding of the construed nature of self leads to less self-focus. This in turn releases capacity for other-focused processes and behaviours. The conditions are formed for the spontaneous appearance of wholesome and prosocial mental states.

*While there are four MGs which encompass the majority of practices employed in contemporary mindfulness programs, these practices may be associated with a wide range of INTs. INTs applied to the four MGs lead to different types of resulting experiential understandings (EUs). Here we detail the EUs for the four specific INTs discussed in this manuscript*.

One way of using the Mindfulness Map is to examine the EUs that may arise when applying different INTs to a particular MG. As an example, we will describe the EUs that may arise when applying the first three INTs within MG1. Practitioners engaged in MG1 practices often experience restlessness, anxiety, boredom, and difficulty to regulate attention. With the INT1 lens (intention to gain experiential understanding how the relationship to experience contributes to mental distress and wellbeing), they can begin to identify the relationship between attention regulation and mental distress. With the INT2 lens (intention to gain experiential understanding of the changing nature of body, mind, and external phenomena), they can begin to notice the constant flow of changing experiences in the present moment and observe the dynamic changing nature of these experiences. The malleability and stability of attention itself can also be understood as changing. This EU enables one to relate to distractions as momentary states, laying the foundations of a non-judgmental attitude further developed in MG2. With the INT3 lens (intention to gain experiential understanding of the relationship between sense of self and mental distress and wellbeing) practitioners can notice the automatic and scattered nature of attention. This can demonstrate, on a rather crude level, how one's agency is limited in controlling somatic and sensory experiences as well as attention to them. Noticing the wandering mind, which is most of the time not subject to one's control, can start to reduce rigid beliefs about the sense of self. The INT4 (intention to gain experiential understanding of how positive and prosocial mental states contribute to wellbeing) is typically not applied when practicing MG1. The limited scope of present moment awareness to somatic experiences may induce non-reactivity, but not necessarily pro-social emotions and attitudes. Critics may even point to the possibility of using the outcomes of M1 practices in ways that contradict INT4; the typical example is “the sniper's mindfulness”—using concentration practices for harming others (e.g., Monteiro et al., 2015).

Another way of using the Mindfulness Map is to examine how a specific INT applied across the four MGs may accelerate insights that can reduce mental distress and increase wellbeing. For example, as mentioned above, applying INT3 (intention to gain experiential understanding of the relationship between sense of self and mental distress and wellbeing) in MG1 enables an initial EU regarding one's limited agency in controlling one's experience. In MG2–MG4 deeper EUs are enabled. The decentering arising in MG2 and further developed in MG4, together with the INT3, enables increased understanding of how the sense of self emerges from craving and avoidance/rejection of present moment experiences and how this relates to mental distress, leading to a rigid sense of self. Non-judgmental acceptance of experience and letting go of craving and avoidance/rejection of experiences enables a more flexible sense of self. The craving and avoidance/rejection patterns can be seen as the “glue” that holds the rigid self together. In addition, the flexible and rigid sense of self relates to the reduction or increase of mental distress, respectively. Applying INT3 with MG3 enables practitioners to further notice that the sense of self is influenced by the wholesome states and pro-social attitudes (e.g., generosity, compassion, gratitude and other similar qualities) and in turn influences the experience of wellbeing. This can further develop into the understanding that the “self,” “other” and the “world” are less rigidly separated than previously assumed. These EUs support the emergence of, and are supported by, additional EUs that can be cultivated by applying INT3 in the more advanced MG4 practices. This allows practitioners to cultivate EUs where the self is understood as a complex phenomenon constructed from changing and interdependent processes and depending on an ever-changing context. As a result, mental distress that is related to a perception of a solid and independent self and can be seen as arising from confusion and misunderstanding and easier to let go of.

## Discussion

In this article we presented a practical classification map of MGs and outlined how these can be taught or practiced with four possible INTs resulting in different EUs. This Map follows several recent attempts to classify meditation and mindfulness practices (Dorjee, 2010; Vago and David, 2012; Schmidt, 2014; Dahl et al., 2015; Garland et al., 2015; Lutz et al., 2015; Grossenbacher and Quaglia, 2017; Khoury et al., 2017; Matko and Sedlmeier, 2019). One influential proposal suggested dividing meditation practices into attentional, constructive, and deconstructive families (Dahl et al., 2015). We resonate with this approach but also recognize that it is more encompassing than our focus on mindfulness practices. In addition, it differs from the Mindfulness Map in its emphasis on underlying cognitive mechanisms of meditation. Finally, none of the previous accounts introduced the dimension of INTs behind mindfulness practices and associated EUs. We will now consider possible applications of the current Map to mindfulness research and teaching.

### Applications of the Map in Mindfulness Research

Current research of mindfulness practices relies mostly on self-report measures to assess mindfulness, drawbacks of which are subject to ongoing discussion, although there have been some recent attempts to design behavioral measures as well (Baer, 2011; Bergomi et al., 2013; Sauer et al., 2013; Levinson et al., 2014; Goldberg et al., 2018a; Wong et al., 2018; Hadash and Bernstein, 2019; Isbel et al., 2020). We suggest a Map, such as the one presented here, may introduce clarity into what aspects of mindfulness practices the different measures are addressing, enabling researchers to better match the tools to the practice they are investigating. For example, the Mindfulness Attention Awareness Scale [MAAS, (Brown and Ryan, 2003)] has been used to study mindfulness-based intervention studies. As its items are mostly assessing difficulties in paying attention (Grossman, 2011), we suggest it mostly assess MG1 and may not capture the full range of expected changes in mindfulness following MG2-MG4. On the other hand, the Five Facets of Mindfulness Questionnaire (FFMQ, Baer et al., 2008) which includes items related to non-judging of inner experience, and non-reactivity toward inner experience may not be appropriate for interventions that mostly rely on MG1 practices. The recently developed Metacognitive Processes of Decentering Scale (Hanley et al., 2020) that attempts to measure how one is able to decenter from internal experience may be mostly relevant for interventions involving MG4 and partially for MG2 practices. In addition, the Map can make more visible some of the gaps across the range of current mindfulness measurement tools and inform the development of new ones. For example, in comparison to MG2-4 there are clearly fewer measures assessing MG3, the Self-compassion scale (SCS) is possibly a rare example (Neff, 2003) targeting the construct of self-compassion which overlaps with the concept of mindfulness.

In addition, the Mindfulness Map explicitly conceptualizes and outlines in practical terms how the INTs may interact with the MG types to result in different EUs. In this way, the Mindfulness Map enables finer-grained assessment of possible modulators (INTs) of intervention outcomes and the associated EUs. No research so far attempted to investigate the modulating role of INTs to outcomes or considered associations between cultivation of various EUs and resulting wellbeing outcomes. The Mindfulness Map enables such distinctions to be drawn and experimentally evaluated. This will necessitate development of new assessment tools to capture different INTs including and beyond those we have outlined and associated EUs. One step to this direction is the current development of the Mechanisms of Contemplative Practice Inventory (Dorjee et al., 2021) which aims to evaluate a range of intentions behind contemplative practice and a progression of modes of existential awareness (EUs). The current Mindfulness Map draws further refined distinctions amongst possible EUs cultivated in secular practice which can be evaluated empirically with tools tailored to assessing these EUs.

The Mindfulness Map also has the potential to enable more accurate cumulative assessments of research evidence regarding particular mechanisms of, or outcomes of, mindfulness practices. Presently, most studies do not distinguish practices in the MG1-MG4 groups and group them together; they also don't take the moderating effects of INTs and possible EUs into account. Consequently, the current systematic reviews and meta-analyses only provide rather crude overarching assessment of the mechanisms and outcomes of mindfulness interventions (e.g., Grossman et al., 2004; Chiesa et al., 2011; Kaunhoven and Dorjee, 2017; Carsley et al., 2018; Garland and Howard, 2018; Goldberg et al., 2018b, 2019, 2021; Wielgosz et al., 2019). This precludes the ability to reach clear conclusions regarding the effects and underlying mechanisms of different mindfulness practices, their interactions and long-term effects (Farias et al., 2016; Schumer et al., 2018; Britton, 2019). Indeed, there have been previously repeated calls for more finer-grained distinctions of mindfulness practices and their intended outcomes (Chiesa and Malinowski, 2011; Davidson and Kaszniak, 2015). For example, recently, two groups conducted a systematic review and a meta-analysis to determine the prosocial effects of mindfulness (Kreplin et al., 2018; Donald et al., 2019). In one case, the analysis concluded that meditation has limited prosocial effects (Kreplin et al., 2018), while in the other, it found medium size effects for the impact of mindfulness interventions on pro-social behaviors (Donald et al., 2019). We suggest such contradictory conclusions may result from collapsing across different MGs and INTs resulting in different EUs.

The lack of these distinctions can also impact on considerations about possible harmful effects of mindfulness. There seems to be an underlying assumption in the mindfulness research that brief mindfulness inductions in beginner practitioners may represent advanced practices such as those found in MG2-4 (e.g., Arch and Craske, 2006; Tan et al., 2014; Norris et al., 2018). For instance, a 5-minute brief-mindfulness induction may instruct participants to pay particular attention to the sensation of their breathing (MG1), but also adapt a particular attitudinal stance (MG2) toward a distracted mind and wandering attention (e.g., to view them as natural, fleeting states of mind, and return attention to their breathing each time a distracting thought, emotion or memory occurred, Tan et al., 2014). For example, in a study that investigated potential morally-negative consequences of mindfulness practice, Schindler et al. (2019), assessed harm-based moral reactions across five studies. In one mindfulness condition participants were asked to listen to a 5-min recording instructing them to concentrate on one's breath and becoming aware of what is happening in the present moment (MG1). In another mindfulness condition participants listened to a 12-min recording which additionally suggested that any emerging feelings and thoughts should be faced in a non-judgmental manner (MG2). Contradictory to their hypothesis, they found that after the brief mindfulness inductions participants were less likely to report morally-aligned intentions. Since it usually takes time and effort to develop the level of concentration needed to sustain a more refined intention, such as understanding the association between relation to experience and mental distress, it is questionable whether such 5 or 12-min practices are sufficient to represent anything beyond MG1 and whether any INT can be applied effectively in such a short time or a relevant EU arise.

These examples suggest an inherent assumption that the various mindfulness practices (of any kind) evoke similar mental states and thus should lead to similar types of psychological or physiological changes regardless of the specific MG employed and the intention of the teachers and students. The Map shows that different combinations of practices and underlying intentions may evoke different mindfulness states. Based on the instructions in Schindler et al. (2019) the participants were engaging in MG1 and MG2 practices without any particular intention instructions. According to the Mindfulness Map, we would not expect prosocial states of mind to be affected by MG1 or MG2 practices alone. Thus, analyzing effects of mindfulness based on MGs, INTs, and EUs, may lead to more precise conclusions regarding the various effects of mindfulness practices.

Finally, the Map can help articulate and support new research questions about mindfulness that are particularly relevant now that the field is moving toward a more refined understanding of mindfulness. Here are some examples: Do different MGs lead to different wellbeing outcomes and physiological changes? Are these modulated by INTs? How do wellbeing outcomes link to EUs? Is training in a range of practices across MGs more likely to result in better wellbeing, or is expertise in particular practices in certain MGs better for fostering wellbeing? Are there individual differences in benefits from different combinations of MGs and INTs? Are explicit ethical INTs (e.g., non-harm) necessary for EUs pertaining to ethical behavior (such as evoking a compassionate stance) to arise? Or do these EUs arise automatically?

### Using the Mindfulness Map to Support Teacher Training and Programme Development

Given that the current teacher training doesn't distinguish across MGs and doesn't explicitly consider the impact of different INTs on teacher EUs and resulting outcomes of mindfulness course participants, the Mindfulness Map provides a framework to open up a focused debate about these factors. Even during a course that lasts for several weeks, there can be a transition between the different MGs of mindfulness and associated INTs. For example, MBSR usually begins with simple body scan (MG1) with the facilitator inviting participants to inquire into the relation between increased attention regulation and lower mental distress (INT1). In later stages of an MBSR program the practice will move to a MG2 practice (e.g., mindfulness of breath) with an invitation to inquire into the changing nature of experience (INT2). Working with the Map may help with a more accurate and comprehensive training of mindfulness teachers, particularly if MG3 and MG4 are explicitly included. This is because most of the practices in MBSR and MBCT are situated in MG1 and MG2 and span mostly INT1 and INT2. The level of expansion to MG3 and MG4 and to the other INTs depends on the EUs the facilitator has cultivated in their personal practice (Kabat-Zinn, 2011), and their ability to lead students in more advanced practices. Teacher training programs should provide opportunities for facilitators to expand their personal EUs through practices that span the full range of the Map, as well as cultivate their skills to facilitate the wider repertoire of EUs in their students through meditation instructions and inquiry techniques that emphasize the various INTs^6^.

In addition, the introduction of the dimension of INTs (including and beyond those discussed in this article) in the Mindfulness Map can facilitate discussions about ethics in western-mindfulness teaching (e.g., Thupten, 2019). Explicit articulation of INTs can, perhaps, help to distinguish between teaching of mindfulness that focuses on INT1 and mindfulness practices taught from a wider perspective of all the INTs. This might be of relevance to current exploration of the potential of mindfulness practices in contributing to societal challenges such as in-group/out group biases, conflict resolution or climate change (Lueke and Gibson, 2015; Tincher et al., 2016; Alkoby et al., 2019; Geiger et al., 2019). For example, practices in MG1 delivered with INT1 (Intention to gain experiential understanding of how the relationship to experience contributes to mental-distress and wellbeing) are unlikely to lead to wider prosocial change, whereas MG2-MG4 delivered with INT3 (Intention to gain experiential understanding of the relationship between sense of self and mental-distress and wellbeing) or INT4 (Intention to gain experiential understanding of how positive and prosocial mental states contribute to wellbeing) may lead to EUs linked to realizing the constructed nature of the sense of self resulting in lessening of in-group identification and out-group rejection. Thus, they may be more suited to lead to prosocial outcomes and behavioral changes, particularly if combined with further context-specific INTs (e.g., climate-friendly behaviour modification).

The Mindfulness Map allows us to understand the limitations of current mindfulness programs and move toward a longer-term perspective on mindfulness practice and its teaching (Dorjee, 2017). For example, since most of the western secular mindfulness programs are based on the MBSR, we can assume that no programs currently incorporate substantial practices from MG4. As mentioned, MBSR cultivates MG1 and MG2, and sometimes incorporates elements of MG3 and MG4 (depending on the training, understanding and capabilities of the teacher), but advanced programs that explicitly develop MG4 in the secular context are rare. There is a growing interest in in-depth secular courses for MBSR graduates (both short programs and long-term), where there is more time to emphasize MG3 and MG4 practices and a broad set of intentions (Levit-Binnun, 2021). A clearer understanding of the range of MGs and INTs underlying various mindfulness practices and programs can assist both teachers and practitioners in making more informed decisions about how to advance their EUs and what guidance to seek. In this way, the Map may facilitate development of a much needed longer-term perspective on mindfulness practice (Dorjee, 2017).

### Limitations of the Mindfulness Map Framework

While the proposed framework has a potential to facilitate more fine-grained distinctions of mindfulness practices, the Mindfulness Map is not intended as a complete framework; rather, it is an initial starting point to open up further discussions about the proposed axes and the MGs and INTs included in the Map. It is possible that the Mindfulness Map needs to be further expanded by additional axes, such as an axis capturing the variety of reasons for engaging in mindfulness practice (Shapiro, 1992; Pepping et al., 2016; Sparby and Ott, 2018). Indeed, it is likely that reasons for mindfulness practice interact with INTs and modulate the resulting EUs further. For example, a practitioner may approach the INTs differently if practicing to reduce their anxiety versus if one practices to realise existential interdependence. Practicing to reduce anxiety would imply a self-focused perspective of mental distress and wellbeing, whereas practicing to realise existential interdependence would involve a more widely encompassing perspective of mental distress and wellbeing including interactions between one's own and others mental distress and wellbeing. Importantly, the proposed framework does not include the full range of meditation practices, INTs and EUs that can be found across contemplative traditions. This means that the map doesn't encompass the full range of phenomenological states and associated experiential knowledge reported in some of these traditions, e.g., states of enlightenment. Finally, the Mindfulness Map proposed in this narrative review requires further extensive empirical research to enable its refinement and to strengthen its empirical grounding.

## Conclusion

In sum, the Mindfulness Map we proposed aims to provide a means to conceptualize and organize the myriad uses of “mindfulness” in current mindfulness-based teaching and research. Describing practices in terms of specific MGs, INTs and expected EUs can contribute to moving the field of contemporary mindfulness research toward more refined understanding of mindfulness practices and their effects and address the prevailing confusions resulting from collapsing across a wide range of mindfulness practices that may be representing different mental states, psychological processes and resulting outcomes. It can also facilitate more focused debate about long-term support of mindfulness teachers and practitioners in their practice, and associated challenges of developing advanced mindfulness programs in the secular context. The proposed Mindfulness Map is aimed as a starting point for further discussion and can be further revised and/or expanded by other axes.

## Author Contributions

NL-B and KA developed the first version of the map. NL-B, KA, and DD developed the final version of the map. All authors contributed to the article and approved the submitted version.

## Conflict of Interest

The authors declare that the research was conducted in the absence of any commercial or financial relationships that could be construed as a potential conflict of interest.

## Publisher's Note

All claims expressed in this article are solely those of the authors and do not necessarily represent those of their affiliated organizations, or those of the publisher, the editors and the reviewers. Any product that may be evaluated in this article, or claim that may be made by its manufacturer, is not guaranteed or endorsed by the publisher.

## Footnotes

^1^The sense of self refers to both conceptual and experiential perception of self.

^2^Slide 21: https://greatergood.berkeley.edu/slides/MindfulnessCompassion-Slides-FINAL-forweb.pdf.

^3^Please note that we use the term “cultivating” here to refer to the mindfulness practice technique rather than the experiential outcome.

^4^While the famous discourse on establishing mindfulness, the satipa??hāna sutta, does not mention pro-social practices as part of the mindfulness inventory, interestingly, the discourse on cultivating friendship, the *metta sutta*, refers to cultivating friendliness as a mindfulness practice.

^5^Although these concepts also appear in much broader contexts in Buddhist discourse, here they are applied in a narrower context, namely, cultivating intentions that are geared toward understanding various mechanisms of wellbeing as they appear in contemporary psychological and scientific discourse.

^6^The same point applies to courses that cultivate compassion (e.g., Mindful Self-Compassion, Neff and Germer, 2018; and Mindfulness-Based Compassionate Living, Van den Brink and Koster, 2015); While these programs seem to emphasize MG3, it remains unclear to what extent MG1 and MG2 are cultivated in these programs, which probably also depend on the level of training of the instructor.
